# Using Procalcitonin to Guide Antibiotic Escalation in Patients With Suspected Bacterial Infection: A New Application of Procalcitonin in the Intensive Care Unit

**DOI:** 10.3389/fcimb.2022.844134

**Published:** 2022-03-14

**Authors:** Xu Wang, Yun Long, Longxiang Su, Qing Zhang, Guangliang Shan, Huaiwu He

**Affiliations:** ^1^State Key Laboratory of Complex Severe and Rare Diseases, Department of Critical Care Medicine, Peking Union Medical College, Peking Union Medical College Hospital, Chinese Academy of Medical Sciences, Beijing, China; ^2^Department of Cardiothoracic Surgery, Erasmus University Medical Center, University Medical Center Rotterdam, Rotterdam, Netherlands; ^3^Department of Epidemiology and Biostatistics, Institute of Basic Medicine Sciences, Chinese Academy of Medical Sciences (CAMS) & School of Basic Medicine, Peking Union Medical College, Beijing, China

**Keywords:** procalcitonin, antibiotics, bacterial infection, critical care medicine, length of ICU stay

## Abstract

**Background:**

Procalcitonin (PCT) is efficient in reducing antibiotic usage without increasing complications for its sensitivity and specificity in detecting bacterial infection. However, its role in guiding antibiotic-spectrum escalation has not been studied. This study was performed to validate the role of PCT in indicating antibiotic spectrum escalation when pathogen results are unknown for ICU patients of suspected bacterial infections.

**Methods:**

This was a single-center retrospective study including patients who were admitted to Peking Union Medical College Hospital from January 2014 to June 2018 for suspected bacterial infections. Patients were divided into “escalation” or “non-escalation” groups according to the change of employed antibiotic spectrum before and after the occurrence of “PCT alert”. The main study endpoint was the length of ICU stay (LIS), and LIS longer than 7 days was defined as “prolonged-ICU-stay (PIS)” while LIS equal to or shorter than 7 days was defined as “non-prolonged-ICU-stay(nPIS)”. Demographics, clinical characteristics, and infection characteristics were compared between patients in the “nPIS” and “PIS” groups. Multivariable logistic regression was used to evaluate independent risk factors for PIS.

**Results:**

Totally, 1109 patients were included, and 654 in the PIS group, other 455 in the nPIS group. Respiratory infection was the main cause in both groups. Patients were older in PIS group than in nPIS group(PIS vs. nPIS: 58.99 ± 16.30 vs. 56.12 ± 15.93 years, P=0.002). The baseline Sequential Organ Failure Assessment (SOFA) score was 11.16 ± 7.33 and 9.73 ± 3.70 in PIS and nPIS groups. Fewer patients received antibiotic escalation in face of “PCT alert” in PIS group (PIS vs. nPIS: 27.68 vs.35.38%, P=0.014). In the multivariable logistic regression model, older age, higher heart rate, not undergoing surgery, higher baseline SOFA score, and not escalating antibiotics in face of “PCT alert” were associated with a prolonged ICU stay. The odds ratio of antibiotic escalation for PIS was 0.582 (95% CI: 0.365, 0.926, P=0.022).

**Conclusions:**

Using PCT to guide antibiotic escalation when pathogen evidence is unavailable could be associated with a shorter length of ICU stay for ICU patients of suspected bacterial infection.

## Introduction

Broad-spectrum antibiotics are recommended for critically infected patients before pathogenic identification as an empirical treatment (Evans et al., 2021). For this group of patients, adjusting the antimicrobial spectrum without pathogen evidence is challenging, not only because of the higher proportion of drug-resistant bacteria in the intensive care unit (ICU) but also due to the importance of applying appropriate antibiotics in the early phase of these patients (De Waele et al., 2018). Thus, physicians need some precise biomarkers to help them identify existing infections and evaluate treatments.

The level of procalcitonin (PCT) is regarded as an indicator of bacterial infection1. Many studies have confirmed that PCT-guided antibiotic de-escalation can diminish the duration and dosage of antibiotics without increasing the risk of complications (Assink-de Jong et al., 2013; Huang et al., 2014; Broyles, 2017; Hohn et al., 2018). The reason why PCT is useful in adjusting antibiotic application for bacterial infection is mainly for its specificity and sensitivity in recognizing inflammation induced by bacteria (Taylor et al., 2017). We can reduce the antibiotic spectrum if PCT decreases as expected or reach a certain value (Assink-de Jong et al., 2013; Huang et al., 2014; Broyles, 2017; Hohn et al., 2018). However, we are also interested in whether undesired PCT reduction indicates uncontrolled infection so that adjustment of antibiotics should be implemented, for example, extending the coverage of spectrum or increasing the dosage. This study was performed to explore the role of PCT in ICU concerning guiding extending spectrum or increasing the dosage of antibiotics.

## Materials and Methods

### Patients

The Institutional Research and Ethics Committee of the Peking Union Medical College Hospital approved this study. Written informed consent was waived since it was a retrospective study.

This was a retrospective study of patients who were admitted to the Department of Critical Care Medicine in Peking Union Medical College Hospital (Beijing, China) for suspected infection. Study period ranged from January 2014 to June 2018.The inclusion criteria were followings: a. equal or older than 18 years; b. length of ICU stay (LOS ICU) longer than 24 h; c. diagnosed with suspected infection; d. having “PCT alert” in ICU stay. Patients who were discharged against medical advice or died were excluded in order to ensure the accuracy of LOS ICU.

### Definitions

#### Suspected Infection

Suspected bacterial infection was defined as receiving antibiotics, and blood or body fluid (e.g. blood, sputum, ascetic fluid, hydrothorax, drainage fluid, etc.) were obtained for culture.

#### PCT Alert

The “PCT alert” was defined as a PCT ≥1.0 ng/mL that was not decreasing at least 10% from the previous day; a single baseline PCT measurement of ≥1.0 ng/mL was also considered to be “alert PCT” (Jensen et al., 2011).

#### Antibiotic Escalation

**Figure 1:**
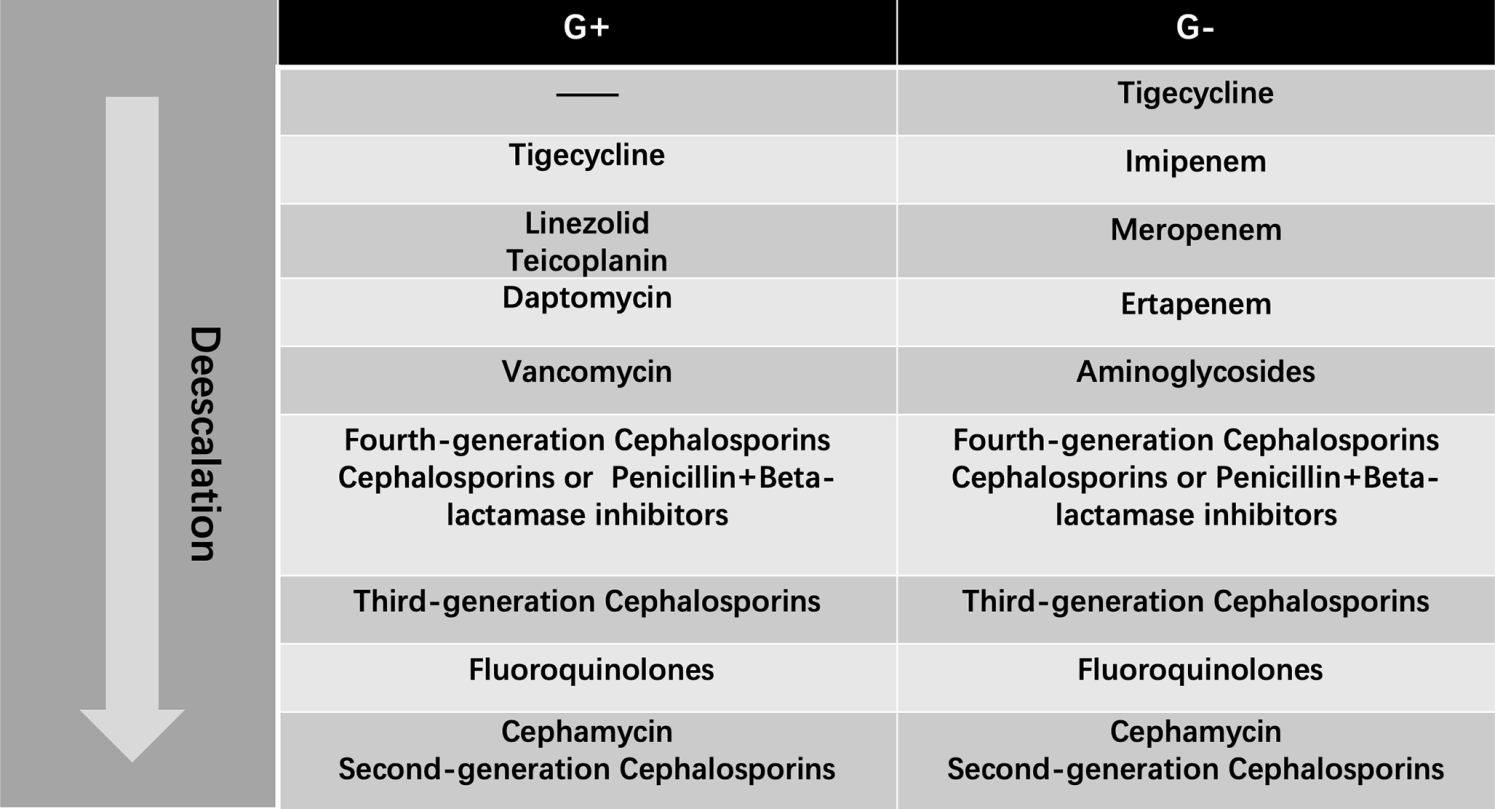
Antibiotic Hierarchy from the Most-Broad-Spectrum (Top) to Least-Broad-Spectrum (Bottom) Antibiotic. G+: Gram-positive Organism Antibiotic Algorithm; G-: Gram-negative Organism Antibiotic Algorithm.

To determine whether the adjustment of antibiotics is “escalation” or not, a “hierarchy system” of antibiotics should be available. In the system, different antibiotics will be ranked according to their spectrums. The broader the spectrum is, the higher the antibiotics are. Considering there is no such consensus on this “hierarchy system”, thus academic publications, local health regulations and a group of senior physicians were consulted to come up with the system in our department. We referred to the document that was released by Beijing Municipal Health Commission concerning antibiotic management. More details of this regulation are presented in **Supplementary File 1**. A published article was referred to as well (Turza et al., 2016). That group of physicians who participated in initiating antibiotic “hierarchy system” contains 4 residents, 4 attending doctors, 2 senior doctors and 2 professors. The final antibiotic hierarchy system is presented in **Figure 1**. The spectrum was divided into two parts generally, G+ for Gram-positive bacteria and G- for Gram-negative bacteria.

Two phases were used to evaluate “escalation” or “no escalation”: within 48 hours before (phase 1) and after (phase 2) the occurrence of “PCT alert”. The information of antibiotics that were used in phase 1 and phase 2 would be compared. The “escalation” was defined as one of the following situations: 1) the maximum grades in “G+” and “G-” spectrums were escalated; 2) the maximum grade in “G+” or “G-” spectrums was escalated; 3) the maximum grades in “G+” and “G-” were unchanged, but the total number of employed antibiotics increased; 4) no adjustment of antibiotic types but the used dosage increased.

### Data Collection

The medical records of all the recruited patients were reviewed to collect the information of sex, age, surgery (with or without), duration of mechanical ventilation (MV), LOS ICU, PCT, white blood cell (WBC) count, maximum daily axillary temperature (Tmax), heart rate (HR), artery lactate, dosage of norepinephrine (NE), Sequential Organ Failure Assessment (SOFA) Score, culture report, and antibiotic information. Our department has a monitoring system that records real-time clinical data from bedside equipment and laboratory examination results. This system is maintained by the Donghua software cooperation through the DtHealth system. The mean arterial pressure (MAP) was not included in analyses because blood pressure would be maintained to normal level by vasopressors or volume infusion, depending on the cause of hypotension,

### Statistical Analysis

Continuous variables were expressed as means ± standard deviations or median (interquartile range), and categorical variables were expressed as absolute values and percentages. The Student’s t-test, the Mann-Whitney U test, the Kruskal-Wallis test, the chi-square or Fisher’s exact tests were used depending on the distributions and type of variables.

A multivariable logistic regression model was constructed to determine the risk factors of PIS. Indicators on the “PCT alert” day. Backward elimination was used in the variable selection. A *P*-value criterion of >0.20 was employed for elimination and <0.10 for retention. Considering laboratory parameters(PCT, lactate, etc.) and Tmax, were collected on 4 different days, the same parameter collected from one patients on different days should be correlated, and including all of them would cause a problem, called collinearity (Greenland et al., 2016). Therefore, only PCT, WBC count, lactate, P/F ratio and NE on the first day in ICU were included into the model. The initial covariates included into the model were followings: age, sex, surgery, baseline SOFA score, culture results, PCT(first day), WBC count (first day), Tmax (first day), P/F ratio (first day), HR (first day), artery lactate(first day), and NE (first day).

All comparisons were two-tailed, and P < 0.05 was required to exclude the null hypothesis. The statistical analyses were performed using IBM SPSS Statistics, Version 20.0 (Armonk, NY: IBM Corp.).

## Results

### Participants

**Figure 2:**
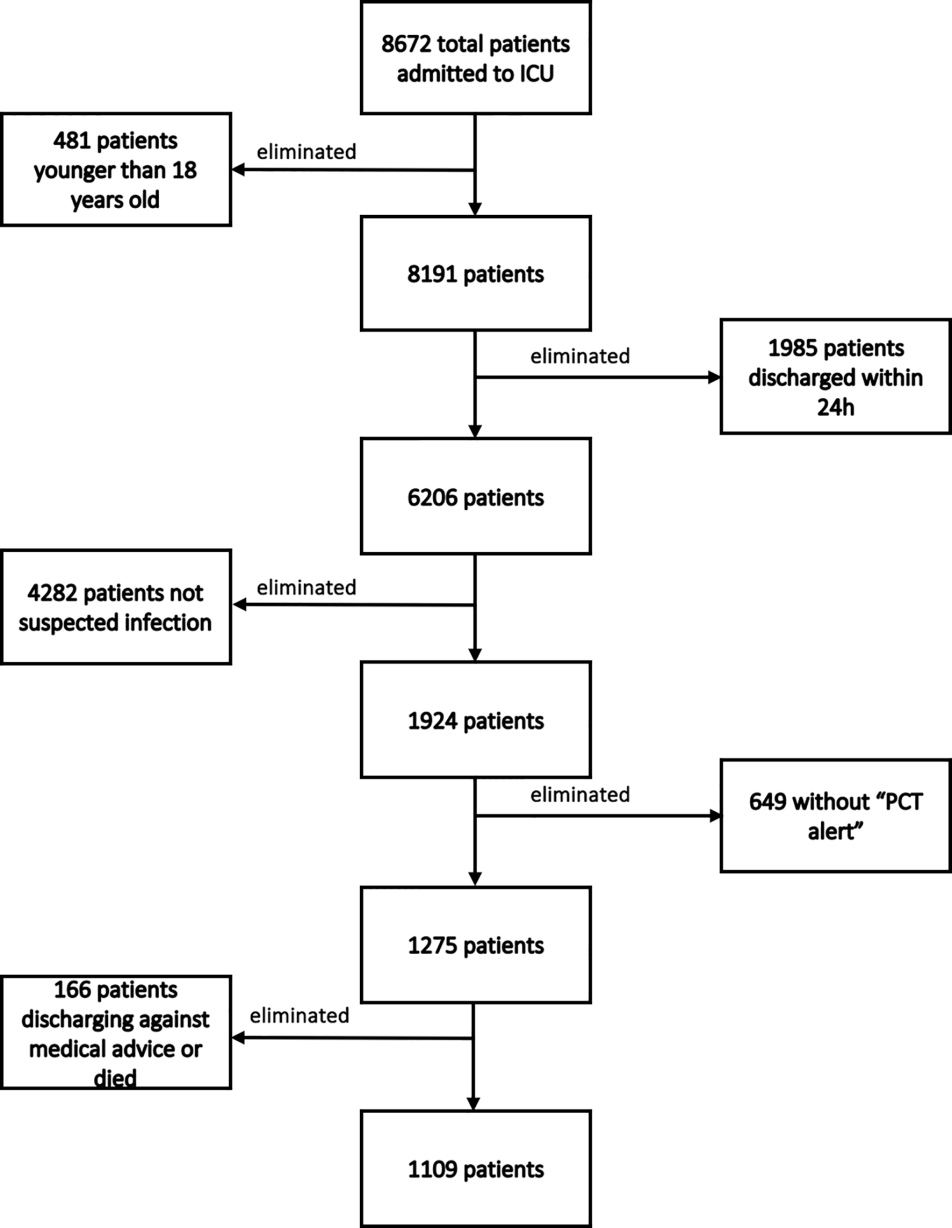
The flowchart of patients’ enrollment.

During our study period, 8672 patients were admitted to our department. A total of 7563 patients were excluded for the following reasons: 481 were younger than 18 years old; 1985 were discharged from the ICU within 24 h; 4282 weren’t suspected infection; 649 did not present a “PCT alert”; and 166 were discharged against medical advice. Finally, 1109 patients were included. The flowchart of patients’ enrollment is shown in **Figure 2**. Among these 1109 patients, 654 belonged to PIS group, while 455 were included in the nPIS group.

### Descriptive Results

The PIS group had older age(PIS vs. nPIS: 58.99 ± 16.30 vs. 56.12 ± 15.93 years, P=0.002), less female patients (PIS vs. nPIS: 34.10% vs. 41.10%, P<0.001) and higher proportion of undergoing surgery(PIS vs. nPIS: 60.55% vs. 45.05%, P<0.001). However, the proportion of antibiotic escalation was lower in PIS group(27.68% vs. 35.38%, P=0.014). More pathogen evidence was obtained later in PIS group than in nPIS group(33.94% vs. 28.13%, P=0.047). The higher SOFA score was also observed in PIS group in contrast to nPIS group(PIS vs. nPIS: 11.16 ± 7.33 vs. 9.73 ± 3.70, P<0.001). There were no differences in infection sites between the two groups (p=0.935) and respiratory infection was the main cause in both groups. No differences were observed between the two groups in PCT values on the 1st, 3rd, 5th and 7th days in ICU. More details of the two groups are presented in **Table 1**.

**Table 1:** Baseline characteristics of the two groups.

		Prolonged N = 654	Non-prolonged N = 455	P
**Age (years)**		58.99 ± 16.30	56.12 ± 15.93	0.002
**Sex (female) n (%)**		223 (34.10)	187 (41.10)	0.021
**SOFA score baseline**		11.16 ± 7.33	9.73 ± 3.70	<0.001
**Escalation** **n (%)**		181 (27.68%)	161 (35.38%)	0.014
**Pathogen results positive n (%)**		222 (33.94%)	128 (28.13%)	0.047
**Surgery n (%)**		396 (60.55)	205 (45.05)	<0.001
**Infection site** **n (%)**	Respiratory	198 (30.28%)	136 (30.55%)	0.935
	Abdominal	128 (19.57%)	85 (18.68%)	
	Urinary	105 (16.06%)	71 (15.60%)	
	Bloodstream	62 (9.48%)	53 (11.65%)	
	Skin soft tissue	48 (7.34%)	37 (8.13%)	
	Unknown	49 (7.49%)	32 (7.03%)	
	Others	64 (9.78%)	41 (8.36%)	
**Tmax** **°C**	1^st^	37.80 ± 0.82	37.87 ± 0.78	0.135
	3^rd^	37.65 ± 0.71	37.54 ± 0.67	0.009
	5^th^	37.57 ± 0.64	37.23 ± 0.59	<0.001
	7^th^	37.48 ± 0.64	37.32 ± 0.57	0.335
**PCT** **ug/L**	1^st^	4.34 (1.52, 17.42)	4.12 (1.45, 13.92)	0.354
	3^rd^	2.96 (1.06, 9.09)	3.69 (1.36, 8.65)	0.236
	5^th^	1.88 (0.71, 5.09)	1.7 (0.64, 5.65)	0.973
	7^th^	1.3 (0.58, 3.59)	1.04 (0.37, 2.87)	0.129
**WBC count** *10^9^/L	1^st^	14.64 ± 8.72	14.69 ± 7.97	0.867
	3^rd^	14.18 ± 8.46	12.89 ± 7.03	0.007
	5^th^	12.54 ± 7.48	10.20 ± 4.85	<0.001
	7^th^	12.36 ± 8.13	10.95 ± 5.28	0.049
**P/F ratio**	1^st^	282.46 ± 138.34	321.70 ± 139.90	<0.001
	3^rd^	323.67 ± 186.41	321.69 ± 123.41	0.423
	5^th^	344.38 ± 202.33	338.79 ± 120.50	0.974
	7^th^	351.20 ± 146.51	294.27.25 ± 20.36	0.38
**Lactate** **mmol.L^-1^**	1^st^	2.00 (1.30, 3.60)	2.05 (1.30, 3.50)	0.548
	3^rd^	1.30 (1.00, 1.90)	1.10 (0.80, 1.60)	<0.001
	5^th^	1.20 (0.90, 1.70)	1.00 (0.70, 1.40)	<0.001
	7^th^	1.20 (0.90, 1.80)	1.60 (1.28, 1.78)	0.861
**NE ug/ (kg.min)**	1^st^	0.26 (0.11, 0.51)	0.17 (0.09, 0.35)	<0.001
	3^rd^	0.22 (0.11, 0.45)	0.16 (0.08, 0.31)	<0.001
	5^th^	0.19 (0.1, 0.36)	0.1 (0.07, 0.26)	0.006
	7^th^	0.18 (0.1, 0.39)	0.3 (0.18, 0.47)	0.16

PCT, Procalcitonin; WBC, white blood cell count; P/F ratio, PaO2/FiO2; Tmax, the maximum body temperature on one day; SOFA, systematic organ function assessment; NE, norepinephrine.

### Antibiotic Escalation

After ran the backward elimination logistic regression, seven covariates were included finally. These covariates were age, surgery, baseline SOFA score, antibiotic escalation, PCT on the first day and Tmax on the first day. The results of the logistic regression are presented in **Table 2**. Older age, not undergoing surgery, higher baseline HR and SOFA score, and not escalating antibiotics were associated with prolonged ICU stay. The odds ratio of antibiotic escalation for PIS was 0.582 (95% CI: 0.365, 0.926, P=0.022).

**Table 2:** Multivariable logistic regression after backward elimination.

Variable	B	Wald	P	OR	95% CI for OR	
					Lower	Upper
**Surgery (Yes)**	-0.704	10.394	**0.001**	0.495	0.323	0.759
**Age**	0.028	15.562	**<0.001**	1.028	1.014	1.042
**SOFA score baseline**	0.155	16.939	**<0.001**	1.168	1.085	1.257
**Antibiotic escalation (Yes)**	-0.542	5.214	**0.022**	0.582	0.365	0.926
**HR**	0.013	5.042	**0.025**	1.013	1.002	1.025
**PCT first day**	0.004	2.340	0.126	1.004	0.999	1.010
**Tmax first day**	-0.196	2.080	0.149	0.822	0.630	1.073

SOFA, systematic organ function assessment; HR, heart rate; PCT, Procalcitonin; Tmax, the maximum body temperature on one day.

**Figure 3:**
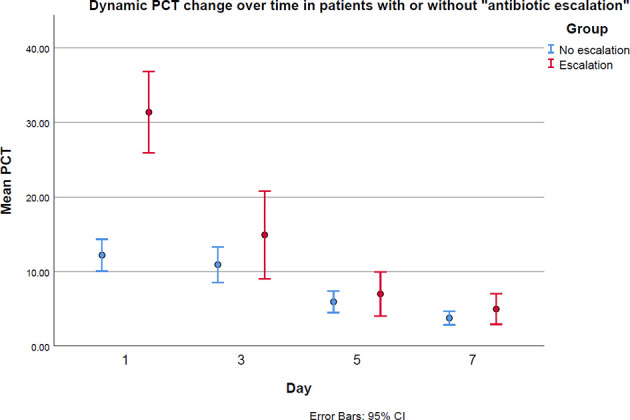
The PCT levels between antibiotic “escalation” and “no escalation” groups on the first, third, fifth and seventh days in ICU.

The dynamic change of PCT in patients with and without antibiotic escalation was plotted and is presented in **Figure 3**. In patients with antibiotic escalation, they had higher PCT value on the first day in ICU, but their PCT values dropped dramatically afterwards. However, the PCT values in patients without antibiotic escalation decreased more stably over time.

## Discussion

The study is the first research aiming at exploring the impact of “antibiotic escalation” on the length of ICU stay for patients with suspected bacterial infection. The most important finding of our study is that escalating empirical antibiotics to a broader spectrum according to “PCT alert”, which is defined as the unideal reduction of PCT or high baseline PCT, is associated with shorter ICU stay. This finding is very meaningful because it gives physicians more references of whether to adjust antibiotics when confronted with unsatisfactory clinical improvement. It is worth noting that the study phase of this research is the time before obtaining clear pathogen evidence. It focuses on the added value of PCT when we have no pathogen reports. There is no doubt that antibiotics should be adjusted in compliance with pathogen evidence if it is available.

Expanding the antibiotic spectrum without certain microbial evidence is often criticized for causing antibiotic overuse (Routsi et al., 2020). However, recognizing pathogen often takes days, and not all pathogen will be successfully cultivated (Idelevich and Becker, 2019). Antibiotic treatment should be initiated as early as possible for ICU patients suspected of bacterial infection (Evans et al., 2021). This study found antibiotic escalation was a positive factor for a shorter ICU stay in the situation of no available culture reports. It is a quite interesting phenomenon, especially as the concerns of antibiotic overuse are growing currently. Several reasons could explain this finding. The high prevalence of multidrug-resistant bacteria(MDRB) in ICU could be the main reason (Routsi et al., 2020). In practice, regular broad-spectrum antibiotics are commonly used as empirical treatments, and that may not strong enough for many patients infected with MDRB. Under this circumstance, escalating antibiotics to a broader spectrum that is against MDRB could benefit patients, for example, escalating from cefixime vancomycin to cover methicillin-resistant Staphylococcus aureus(MRSA). Our hospital is a tertiary hospital, and many patients in our department are transferred from other hospitals and have long-term antimicrobial application. Therefore, the proportion of multidrug resistant bacteria may be high. The special properties of these recruited patients might cause difficulty in extending our findings to patients without long history of using antibiotics. However, antibiotic escalation should be considered when “PCT alert” presents after initial treatment, if patients have higher probabilities of multidrug resistant bacteria.

Besides, the severity of the infection could also influence the impact of antibiotic escalation. As we see from **Figure 3**, the mean baseline PCT values in both “escalation” and “no escalation” groups are very high. High PCT levels have been proved to be associated with increased mortality (Covington et al., 2018). Therefore, patients enrolled in this study are severely sick and could benefit more from an intensive antimicrobial therapy than mild patients do because they are at much higher risk of death than mild patients are, if not strong enough antimicrobials are used early (Evans et al., 2021). That could be another reason to explain why escalating antibiotics reduces ICU stay, even though pathogen evidence is unknown. In view of these patients’ special properties illustrated above, which are of higher probabilities of MDRB and serious sickness, it is suggested to upgrade antibiotics if PCT levels have not shown satisfactory reduction. Nevertheless, for patients who have no pre-existing long-term antibiotics application or not severely ill, waiting for culture reports to adjust antibiotics could be a good solution, but future research is necessary to discuss the impact of antibiotic escalation stratified by patients’ risk levels.

Despite the baseline of PCT being higher in escalation group, it dropped dramatically over time, as shown in **Figure 3**. Xiao et al. (2021) have proved dynamic changes in PCT have a certain role in predicting the therapeutic effect and prognosis of anti-infection treatment. That is why “PCT alert” has its role in evaluating the effects of antibiotic treatment. If physicians confront with non-decreased even increased PCT after empirical treatments start, they should carefully re-check patients’ medical records and consider whether to change antibiotic spectrum. It is worth noting that escalating antibiotics should not be done only based on “PCT alert”. Adjusting anti-infection treatment without pathogen evidence is always challenging, and more aspects being considered, more chance of selecting the suitable drugs.

Different from the findings of our study, Jensen et al. (2011) proved that antimicrobial spectrum escalation guided by PCT prolonged the patients’ ICU stays and increased the possibility of organ-related harm. However, there was an important inherent design flaw in this study: the physicians were not double-blinded to the PCT values, which may have led to bias because physicians tend to be “overcautious” if they are alerted to an abnormal PCT level. This means that physicians’ decisions may have been unfairly influenced in the “PCT intervention” group. However, all of the patients included in this study had PCT tests, and the results were informed to their physicians. Hence, bias caused by non-double-blinded to the PCT values does not exist in our study.

Another interesting finding in this study is surgical patients seem to have a lower risk of staying in ICU for longer than 7 days. It is not easy to understand because surgery should add the probability of prolonged ICU stay. One of the reason to explain it is our department is a surgical ICU ward, and patients admitted to our department for surgery usually have less severe health conditions than patients who are admitted for non-surgical reasons. Most of the no-surgical patients were transferred from department of internal medicine and have longer pre-ICU in-hospital stay than surgical patients. It could lead to a high probability of nosocomial infections and increase the length of ICU stay (Nakamura and Tompkins, 2012).

### Limitations

There were some limitations to our study. First, we only enrolled patients with “PCT alert”, and that limits our findings only applicable to patients have high enough PCT levels. Besides, most of the patients who were included in this study were relatively severe. Their baseline PCT levels and SOFA scores were quite high, indicating they were at high risk of mortality. Whether the benefits of antibiotic escalation could be observed in patients not at that high risk needs future studies accounting for risk stratifications. Additionally, we did not establish very strict inclusion criteria, and included patients were heterogeneous from baseline characteristics to health-related conditions. Escalating antibiotics is a process balancing between benefit and loss. For some specific groups of patients, they may benefit more and lose less from the escalation than others. Analyzing all these patients together may blur the actual situations in patients with different characteristics. In the future, specific studies accounting for more aspects could fill the gap. Finally, as a retrospective observational study, it is inevitable that many confounders exist (Kaji et al., 2014). Despite we controlled some of them by constructing multivariable logistic regression model, it is impossible to consider all potential confounders. Therefore, the causal relation between antibiotic escalation and shorter ICU stay is not as convincing as a randomized controlled trial.

## Conclusions

Procalcitonin-guided antibiotic escalation in the intensive care unit decreases patients’ length of ICU stay. When pathogen evidence is unavailable, and patients have not shown PCT improvement after empirical antibiotic application, escalating the antibiotics to a broader spectrum could be a solution, especially for patients at a high risk of infection with multidrug-resistant bacteria and death.

## Data Availability Statement

The raw data supporting the conclusions of this article will be made available by the authors, without undue reservation.

## Ethics Statement

The studies involving human participants were reviewed and approved by The Institutional Research and Ethics Committee of the Peking Union Medical College Hospital. Written informed consent for participation was not required for this study in accordance with the national legislation and the institutional requirements.

## Author Contributions

YL conceived and designed the study, interpreted data, and helped draft the manuscript. XW and HH participated in the study conception and design, recruited patients, collected data, performed the statistical analysis, interpreted the data, and drafted the manuscript. QZ participated in technical support and data collection. LS contributed to the critical review of the manuscript. participated in data collection. GS assisted us in performing the statistical analysis. All authors contributed to the article and approved the submitted version.

## Funding

Excellence Program of Key Clinical Specialty of Beijing in 2020, critical care medicine (zk128001).

## Conflict of Interest

The authors declare that the research was conducted in the absence of any commercial or financial relationships that could be construed as a potential conflict of interest.

## Publisher’s Note

All claims expressed in this article are solely those of the authors and do not necessarily represent those of their affiliated organizations, or those of the publisher, the editors and the reviewers. Any product that may be evaluated in this article, or claim that may be made by its manufacturer, is not guaranteed or endorsed by the publisher.

## Abbreviations

PCT, procalcitonin; ICU, intensive care unit; nPISs, non-prolonged ICU stays; PISs, prolonged ICU stays; WBC, white blood cell; OR, odds ratio; CI, confidence interval; G+, gram-positive; G-, gram-negative; LOS ICU, length of ICU stay; MV, mechanical ventilation; Tmax, maximum daily temperature; NE, norepinephrine; SOFA, Sequential Organ Failure Assessment; P/F ratio, the ratio of the PaO_2_ (arterial oxygen partial pressure obtained from an arterial blood gas) to the FiO2 (fraction of inspired oxygen).
