# Supplementary material for: Using Procalcitonin to Guide Antibiotic Escalation in Patients With Suspected Bacterial Infection: A New Application of Procalcitonin in the Intensive Care Unit
Source: Front Cell Infect Microbiol. 2022 Mar 14;12:844134. doi: 10.3389/fcimb.2022.844134 (PMC8964283; doi:10.3389/fcimb.2022.844134)
Supplement: Supplementary file 2 [file Table_2.docx]

Additional file 1:

This is a file describing the general classification of different antimicrobials released by Beijing Municipal Health Commission in order to manage antibiotic application in hospital. In this document, all antibiotics were labelled as “no restriction”, “restriction” or “only under very special condition” according to their property. “No restriction” means every doctor has right to prescribe the drugs. “Restriction” implicates only superior doctors have the prescriptive right of the drugs. “Only under very special condition” indicates doctors have to ask for instructions from the head of department to prescribe these drugs, giving adequate reasons for his/her medical decisions.
